# Mental health nurses' knowledge and attitudes toward clozapine management protocols challenges and implications for clinical practice: A narrative review

**DOI:** 10.3934/publichealth.2026004

**Published:** 2026-01-06

**Authors:** Polyxeni Mangoulia, Maria Topi

**Affiliations:** 1 Laboratory Nursing Counseling Support and Psychoeducation of Patients with Mental Illness and Caregivers, Faculty of Nursing, National and Kapodistrian University of Athens, Greece; 2 Eginitio Hospital, 1^st^ Department of Psychiatry, National and Kapodistrian University of Athens, Greece

**Keywords:** clozapine, administration protocols, side effects, nurses, mental health nurses, knowledge, attitudes

## Abstract

Clozapine is an atypical antipsychotic primarily used in cases of treatment-resistant schizophrenia, showing significant clinical efficacy. However, a major challenge in its administration is the requirement for systematic monitoring, which necessitates nurses to possess documented knowledge and skills. In this study, we aimed to synthesize nurses' knowledge and attitudes toward clozapine administration protocols and the importance of education for ensuring safe clinical practice. A literature review spanning the past decade was conducted using PubMed, Google Scholar, and Scopus databases, focusing on English terms related to nurses' understanding of clozapine administration protocols and the associated needs and implications in clinical settings to construct a comprehensive narrative review on this subject matter. Findings revealed that approximately half the nurses surveyed reported inadequate knowledge regarding the side effects of clozapine, leading to significant implications for patient monitoring, treatment, and medication adherence. Nurses with higher levels of education exhibited greater knowledge levels. Factors such as lower education levels, limited clinical experience, high workload, fatigue, and blind adherence to medical instructions were found to influence nurses' knowledge and attitudes. Training programs were shown to reduce stress, enhance nurses' knowledge and confidence, and foster trust in patient care. Nurses were identified as playing a crucial role in the safe administration of clozapine, both within hospital settings and in the community. Education was highlighted as a key factor in improving knowledge levels and shifting attitudes among nurses and patients toward the safe use of clozapine.


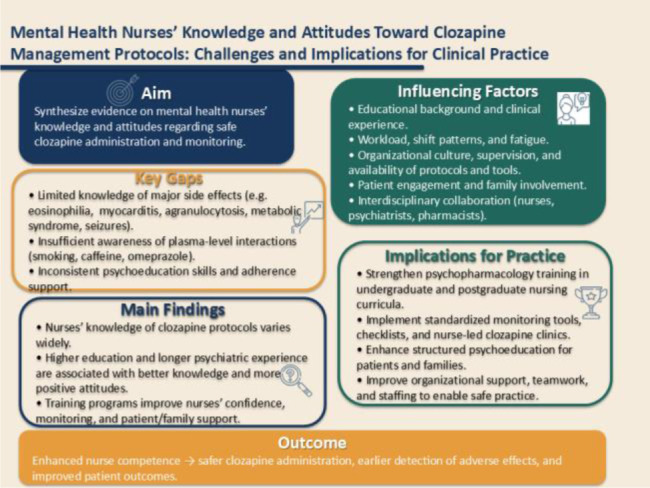


**Graphical abstract.**

## 1. Introduction

Clozapine is an atypical antipsychotic indicated for the treatment of psychotic disorders and schizophrenia. It has been proven to be an effective first-line treatment for refractory schizophrenia [1]. Additionally, clozapine is sometimes used off-label to treat bipolar disorder, depressive disorder, borderline personality disorder, tardive dyskinesia, and tardive dystonia [2]. Beyond reducing the positive symptoms of schizophrenia, clozapine has demonstrated effectiveness in decreasing aggression, suicidality, long-term mortality, relapses, and recurrent hospitalizations, ultimately improving overall functioning [3],[4].

Clozapine has a half-life of approximately 12 hours, reaches maximum plasma levels within 2 hours, and achieves a stable plasma concentration in less than one week [5],[6]. Evidence indicates that clozapine can reduce extrapyramidal symptoms induced by other antipsychotics [1]. Although it is not typically used as a first-line antipsychotic, approximately 60% of individuals who do not respond to other antipsychotics experience significant clinical improvement with clozapine [7]. Despite its efficacy, initiation of clozapine treatment often occurs four to five years after disease onset, and its administration rates remain low [1],[8],[9].

One reason clozapine is not commonly used as a first-line treatment for schizophrenia is its association with serious and potentially life-threatening side effects. Mandatory monitoring of the patient's laboratory values and clinical status during the initial treatment period is essential to detect these dangerous side effects [10]. Additionally, a lack of training among healthcare professionals in prescribing clozapine, as well as limited experience in administering and monitoring patients, presents significant barriers [11].

Side effects of clozapine treatment can range from mild to severe and include salivation, drowsiness, constipation, nausea, vomiting, weight gain, agranulocytosis, eosinophilia, thrombocytopenia, myocarditis, seizures, hyperlipidaemia, intestinal ischemia or obstruction, liver dysfunction, acute liver failure, and metabolic syndrome [12]. These adverse effects may negatively impact patients' adherence to treatment [13].

Strict protocol-based monitoring is essential for individuals initiating clozapine treatment. During the first 18 weeks, patients should undergo weekly laboratory tests, blood work, and monitoring of vital signs, including blood pressure, pulse, temperature, and weight [5]. Specialized training for healthcare professionals in safe clozapine administration protocols can further enhance patient safety and treatment outcomes [14].

Mental health nurses play a crucial role in monitoring and early detection of clozapine side effects, as well as in educating patients and their families. Specialized clozapine clinics, often led by trained nurses, exist worldwide. However, there is a gap in the training of mental health nurses regarding clozapine administration protocols and side effect management [5],[12].

In this narrative review, we aim to synthesize the knowledge, perceptions, and experiences derived from clinical practice of mental health nurses concerning the safe administration of clozapine and the monitoring of its side effects. Education and expertise are essential for mental health nurses to effectively fulfil their roles, including providing psychoeducation to patients and families to ensure treatment safety, build trust, and promote adherence.

## 2. Materials and methods

We provide a concise synthesis of the literature pertaining to mental health nurses' knowledge and attitudes toward the safe administration, management protocols, and side effects of clozapine. Furthermore, we underscore the need for comprehensive educational initiatives targeting nurses in this domain and examine the resultant advantages for their professional responsibilities, clinical practice, and patient care outcomes.

We focused on studies published within the past decade (2014–2025). The search was performed utilizing the PubMed, Google Scholar, and Scopus databases. To enhance the specificity of the search results, keywords such as mental health nurses, nurses, knowledge, clozapine, administration protocols, management protocols, side effects, attitudes, training, and education were employed. The criteria for paper selection were deliberately designed to be less restrictive to promote a broad conceptual contribution and thematic relevance. A comprehensive and flexible search strategy was employed to capture a diverse range of perspectives.

In this review, we encompassed original research studies, systematic reviews, and meta-analyses published in the English language. To ensure a focused scope, studies involving healthcare professionals other than mental health nurses were excluded. Additionally, opinion articles, editorials, non-English publications, and studies lacking accessible full texts were omitted. The final inclusion of articles was determined based on their scientific rigor, clinical relevance, and congruence with the objectives of the review.

## 3. Results

### 3.1. Identifying shortcomings and barriers

Research in the international literature has highlighted the challenges and, in some cases, the inability of mental health nurses to effectively address knowledge inquiries related to the administration and adverse effects of clozapine. Significant gaps have been identified in mental health nurses' understanding of clozapine side effects [4],[5],[13]. A survey of 100 mental health nurses in a city in Egypt revealed insufficient knowledge regarding the pharmacokinetics and pharmacodynamics of clozapine, as well as nursing practices during its administration. Notably, it has been observed that mental health nurses often administer antipsychotic medications to their patients regardless of their level of expertise with these drugs. Additionally, 81% of respondents reported having sufficient awareness of the indications for which clozapine can be prescribed [14].

The level of education appears to be a crucial factor contributing to mental health nurses' lack of understanding regarding clozapine. Unfortunately, nurses trained through two-year programs do not receive pharmacology and psychopharmacology courses in their undergraduate curriculum, unlike those trained through four-year programs. Other relevant concerns include the scarcity of continuing education programs in hospitals and the presence or absence of extensive clinical experience in similar roles. Clinical expertise and years of work in psychiatric clinics and hospitals improve the ability to identify and manage clozapine side effects [15]. Finally, nurses are frustrated by rotating shifts, heavy workloads during their shifts, and a lack of motivation for professional development. The factors listed above appear to deter their desire for further growth [14].

In addition to nurses' level of competence, several significant barriers to clozapine administration have been identified. One major obstacle is the availability of specialized clinics where clozapine and its formulations are administered by trained and qualified healthcare professionals. Another challenge is the requirement for systematic monitoring of laboratory values for certain medications, which is more frequent at the start of treatment and less frequent thereafter. This need for regular monitoring demands patient awareness, agreement to pharmacological adherence and monitoring, and the development and implementation of psychoeducation protocols by mental health specialists.

Equally noteworthy is the lack of tools for recording and evaluating the side effects of clozapine and other antipsychotic medications in nursing clinical practice [14]. Similar technologies are also absent in nursing education and clinical practice despite their potential to significantly advance nursing research and optimize patient care. Despite these deficiencies, obstacles, and daily challenges, mental health nurses generally respond adequately to clozapine administration, particularly in clinical settings. Unfortunately, this effectiveness largely depends on the operation of a medically centered healthcare system in which nurses follow and implement medical orders [3],[14].

### 3.2. Nurses' knowledge and attitudes toward clozapine adverse effects

Myocarditis is a significant adverse effect of clozapine, affecting approximately 3% of patients and typically occurring within the first four weeks of treatment. The mortality rate ranges from 20% to 64% [16]. According to the literature, awareness and recognition of myocarditis symptoms remain quite low [5]. Monitoring for clozapine-induced myocarditis requires assessment of signs and symptoms, along with diagnostic testing.

Clozapine has a potentially fatal side effect known as agranulocytosis. To prevent mortality, stringent blood testing protocols are implemented weekly for the first 18 weeks, followed by testing every other week for the subsequent 52 weeks. Approximately one-third of nurses were aware of clozapine-induced agranulocytosis [17]. In addition to frequent laboratory monitoring, nurses should observe and document symptoms resembling those of the flu or other illnesses. This information, combined with laboratory results, can support the prompt discontinuation of clozapine if necessary.

Recognizing the potential for developing metabolic syndrome and diabetes mellitus is a side effect that approximately one-third of nurses seem to understand. Although clozapine may not cause metabolic syndrome as an immediate life-threatening side effect, it is essential to be aware of its occurrence. This awareness enables healthcare providers to offer patients information and psychoeducation aimed at preventing, managing, and maintaining their physical health [18].

Furthermore, 20%–30% of nurses are aware of the risk of clozapine-induced seizures, which are often associated with elevated plasma drug concentrations [5]. These seizures are considered adverse events that do not warrant discontinuation of clozapine treatment or pose a life-threatening risk. Mental health nurses' knowledge of the medication and patient education enable them to manage seizures effectively and respond appropriately by adjusting or discontinuing the medication if plasma concentrations increase.

Approximately 50%–75% of mental health nurses are aware that clozapine therapy can cause constipation. Although it is not an immediately life-threatening adverse effect, it should be recognized and monitored during clozapine treatment due to the risk of intestinal obstruction and paralytic ileus, which can be fatal [5].

Finally, only one in every four nurses is aware of how patients' smoking behaviors affect their plasma clozapine concentrations [5]. One in every three mental health nurses understand the effects of coffee or omeprazole use. Omeprazole lowers clozapine plasma concentration, while coffee and all caffeine-containing beverages increase it. Adjusting patients' smoking or coffee intake, as well as their consistent use or avoidance of omeprazole, is an important aspect of psychoeducation during clozapine treatment [19],[20].

Mental health nurses generally possess greater knowledge about clozapine administration and its side effects, likely due to their employment and clinical experience. However, a survey of mental health nurses in Belgium revealed that only one in four could correctly answer at least half the questions regarding clozapine's adverse effects, despite their experience in psychiatric wards. Nevertheless, 70% were aware of the circumstances under which clozapine is administered.

In the same survey, barely half the participants were aware of agranulocytosis, and only 15.3% recognized its clinical signs and symptoms. It is essential for nurses to identify these clinical indicators promptly. In this study, only half the participants were informed about clozapine's side effects, including constipation. In contrast, just one in five nurses correctly identified the dose-dependent risk of seizures associated with clozapine. Approximately one-third of nurses were aware of metabolic syndrome as a side effect of clozapine and could recognize it clinically. Additionally, 60% of participants were unaware of the protocol for monitoring laboratory blood tests at the initiation of clozapine therapy [5].

A survey of mental health nurses at an Egyptian hospital revealed that more than two-thirds were unaware of clozapine's cardiovascular and urinary tract side effects, as well as the drug's risk of inducing metabolic syndrome. Similarly, just over half were fully aware of the dangers of neutropenia and agranulocytosis. More than two-thirds of the nurses had sufficient information to obtain a comprehensive, adjusted history of patients treated with clozapine and to monitor their vital signs at the beginning and end of treatment. However, they lacked expertise in providing psychoeducation related to clozapine treatment for patients and their families [14].

### 3.3. Nurses' and patients' attitudes and views toward clozapine

A comparative assessment of the knowledge and attitudes of patients treated with clozapine and health professionals revealed that both groups considered clozapine to be a fairly effective medication. However, neither group regarded clozapine as highly safe, with patients perceiving it as safer than mental health specialists did. Regardless of clinical experience, mental health experts reported a predominantly positive attitude toward the efficacy and necessity of clozapine. Those with the most clinical experience also demonstrated a better understanding of its potential adverse effects [1].

People taking clozapine seem to be more concerned about certain side effects than health professionals. Notably negative views toward clozapine have been documented, with adverse effects such as excessive salivation, constipation, drowsiness, and delayed psychotic recovery reported. In contrast, most health professionals are primarily concerned with side effects like agranulocytosis, the risk of developing diabetes mellitus, and the cardiovascular effects of clozapine [1],[21].

Most nurses recognize and report that clozapine underprescribing results from challenges and obstacles faced by healthcare professionals and patients. Clozapine requires strict medication adherence and regular monitoring, along with significant daily side effects that can make it difficult for patients to adjust. Additionally, mental health nurses encounter substantial gaps in training and infrastructure [21].

Patients' positive attitudes and perceptions of clozapine arise from their belief that it is an effective treatment, and they are willing to invest time and effort in its use. Conversely, negative attitudes pose a significant barrier [22]. This issue profoundly affects patients' lives in the short and long term. Therefore, the importance of adequate infrastructure and properly qualified mental health professionals is underscored.

### 3.4. The significance of educating nurses and patients

Serious adverse events and the need for regular laboratory test follow-up during clozapine treatment are two of the most reported reasons in the literature for poor pharmacological compliance or treatment discontinuation [23]. In this context, mental health nurses play a crucial role in patient care, symptom management, side effect monitoring, and psychoeducation [24]. This underscores the importance of healthcare professional training, as well as the development of clinical tools and evidence-based guidelines for routine clinical practice to ensure safe administration. Health and mental health experts emphasize the need for standardized protocols, comprehensive training, sufficient time allocation, and the availability of specialized clinics for clozapine administration to safely manage, monitor, and support patients [21].

Researchers have identified significant deficiencies in nurse training within the field of psychopharmacology [25]. Typically, education is confined to undergraduate coursework and, at later stages, brief seminars and lectures. Research conducted in the Western Cape revealed that only 38 of the 90 nurses surveyed had received psychopharmacology training [26]. Beyond training, mental health nurses also face a considerable shortage of tools for assessing adverse reactions in their clinical practice [27].

In collaboration with psychiatrists, mental health nurses play a crucial role in monitoring and documenting the clinical status of patients treated with clozapine, as well as identifying and managing adverse reactions. This role is essential for ensuring medication adherence and for the effective treatment and prevention of relapse in these individuals [28]. Consequently, investing in psychopharmacology education for nurses is imperative, as the foundational training provided at the undergraduate level is often limited [29],[30]. A review of psychopharmacology training programs for mental health nurses demonstrated a significant increase in knowledge and confidence regarding the safe administration of clozapine, as well as the monitoring and management of adverse events. This training also enhanced psychoeducation and support for patients receiving clozapine and their families [5],[30].

Finally, nurses should receive comprehensive psychopharmacology training at the undergraduate and postgraduate levels. This shift moves patient care away from a strictly medical-centered model toward a more psychosocial and holistic approach. It influences clinical recommendations and judgments in each case, emphasizing the individual's well-being [31],[32].

### 3.5. Effectiveness of patient-centred nurse training

Studies of clozapine clinics managed by trained nurses demonstrated a higher number of clinic visits, increased utilization of community psychiatry services, and effective monitoring of caffeine and nicotine intake alongside clozapine medication. Furthermore, the establishment and operation of clozapine clinics by appropriately qualified nurses led to lower costs compared to clinics overseen by physicians [7].

Clinics with multidisciplinary teams that include psychiatrists, pharmacists, and specially trained mental health nurses tend to be equally effective in promoting clozapine compliance and retention [3],[15]. Counseling patients by appropriately qualified healthcare personnel helps identify side effects and support continued adherence to treatment [24].

Mental health nurses with a four- or five-year nursing diploma demonstrated higher levels of knowledge than those with only two years of training. This highlights the importance of integrated educational programs that include comprehensive pharmacology and psychopharmacology instruction in their curricula [16].

Nurses trained in psychiatric nursing demonstrate superior knowledge and performance in psychopharmacology, including the efficacy of antipsychotic drugs, their adverse effects, and treatment protocols [27],[28],[32]. As frontline providers of patient care, it is essential that nurses remain vigilant and well-informed about potential side effects and drug interactions [32].

### 3.6. Synthesis of findings

Our findings collectively indicate that nurses' knowledge and attitudes toward clozapine administration are shaped by a dynamic interaction of educational, organizational, and patient-related factors. This synthesis underscores that safe clozapine practice depends not only on individual knowledge but also on workplace conditions, institutional support, and interdisciplinary collaboration.

#### 3.6.1. Conceptual synthesis

**Figure 1.**
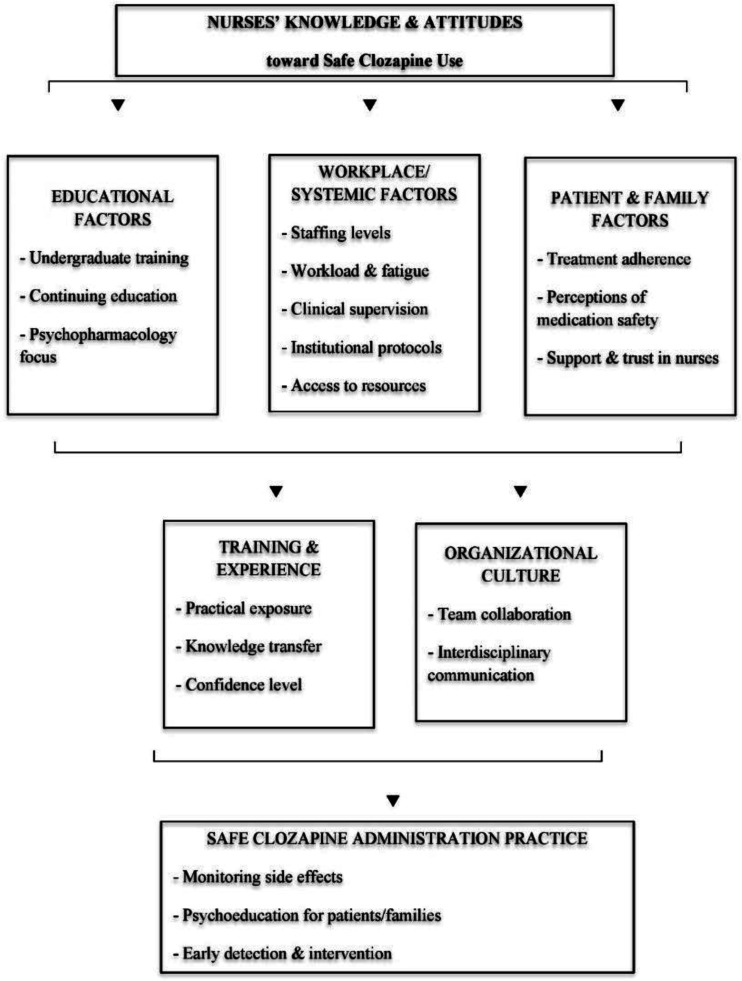
Conceptual model of factors influencing nurses' knowledge and attitudes toward safe clozapine administration.

To capture the interconnections between these dimensions, Figure 1 presents a conceptual model illustrating how education, professional experience, systemic factors, and patient engagement converge to influence nurses' confidence and competence in clozapine administration. Educational background and continuing training form the foundation of knowledge and confidence. Workplace and systemic conditions, such as staffing levels, supervision, and access to clinical protocols, determine whether this knowledge can be applied effectively in daily practice. Patient and family factors, including treatment adherence and perceptions of medication safety, further shape nurses' attitudes and their capacity to provide psychoeducation. At the organizational level, collaboration, communication, and leadership culture reinforce or hinder the translation of knowledge into safe practice. Collectively, these elements lead to the overarching outcome of safe clozapine administration, characterized by accurate monitoring, early detection of side effects, and effective patient support.

Figure 1 therefore encapsulates the multi-level framework emerging from the literature: Nurses' knowledge and attitudes are not isolated traits but part of a broader, interdependent system linking education, context, and care quality.

#### 3.6.2. Implications for practice

Building on this synthesis, several key implications for clinical and educational practice emerge. These implications reflect the main thematic areas identified throughout the review, education and training, clinical monitoring, workplace support, psychoeducation, and organizational policy, and highlight practical actions required to strengthen nursing competence and patient safety.

Table 1 summarizes these implications for practice, providing a concise yet comprehensive overview of how mental health nurses and institutions can translate the evidence into everyday clinical decision-making. It organizes the implications across five domains, educational, clinical, systemic, patient-centered, and policy-level, each grounded in the findings presented earlier. The table thus functions as a bridge between evidence and application, showing how the themes identified in the literature can inform curriculum design, institutional strategies, and policy development. Through this synthesis, the review underscores that improving safe clozapine administration requires a coordinated, multi-level approach, combining individual professional growth with system-wide organizational support.

**Table 1.** Summary of key implications for practice in safe clozapine administration by mental health nurses.

**Domain**	**Key Insights from the Review**	**Practical Implications for Nursing Practice**
Education and Training	Nurses' knowledge of clozapine protocols is often limited, especially among those with shorter educational programs or minimal psychopharmacology exposure.	• Integrate psychopharmacology and medication safety modules into undergraduate nursing curricula. • Implement regular continuing education and simulation-based workshops focused on clozapine monitoring and side-effect management.
Clinical Practice and Monitoring	Nurses play a pivotal role in identifying adverse effects and ensuring adherence but often lack structured protocols or decision-support tools.	• Develop standardized checklists and monitoring tools for clozapine side effects. • Encourage nurse-led monitoring clinics and shared decision-making with psychiatrists.
Workplace and Systemic Support	High workload, shift rotations, and lack of supervision hinder safe medication administration.	• Optimize staffing and workload distribution in psychiatric units. • Establish clear institutional protocols and mentorship systems to support nurses' clinical decision-making.
Psychoeducation and Patient Engagement	Patient understanding and attitudes strongly influence adherence to clozapine treatment.	• Train nurses in communication and psychoeducation strategies. • Provide patients and families with accessible information on benefits, side effects, and required monitoring.
Organizational and Policy Level	Multidisciplinary collaboration and organizational culture shape nurses' confidence and clinical performance.	• Promote interdisciplinary teamwork and empower nursing roles in medication safety policies. • Encourage health authorities to fund nurse-led psychopharmacology training initiatives.

### 3.7. Limitations of the study

The primary limitation is the narrative review technique. A narrative review, unlike a systematic review, does not analyze the quality or bias of the included research, which may influence the findings. Furthermore, our broad scope, which focuses on an overview rather than in-depth investigation or precise responses, may result in studies that complicate synthesis. Narrative reviews are descriptive and exploratory rather than quantitative/qualitative synthesis or intervention effectiveness. Though possibly less resource-intensive than systematic reviews, they necessitate significant effort and are not suitable for very detailed queries. However, this broad approach met our purpose of laying the groundwork for future, more targeted research.

## 4. Conclusions

Clozapine is a relatively old drug; however, it is underprescribed. While researchers have focused on the side effects and challenges of administering and monitoring patients treated with clozapine, few have examined nurses' roles and competencies in providing support for its safe administration.

Barriers to initiating clozapine treatment have been identified at three levels. The first level involves nurses' knowledge and perceptions regarding drug administration and side effect management. The second level pertains to hospital and clinical infrastructure that is inadequate to support healthcare professional training and follow-up examinations during clozapine therapy. Finally, barriers exist at the patient and family level, stemming from a lack of awareness, fear of adverse effects, and insufficient information and support from mental health specialists.

According to available research, fewer than one in every two nurses has a thorough understanding of clozapine administration protocols and its side effects. This highlights the need for specialized undergraduate training in administration procedures, pharmacology, and psychopharmacology. Mental health nurses should also continue their clinical training to enhance their expertise.

By developing safe and well-designed protocols as tools for nurses and raising their awareness through training, they contribute to their patients' psychoeducation and support. The goals are good medication adherence, optimal functioning, and reduced relapse rates.

Medication management remains one of the major responsibilities of mental health nurses. For this reason, proper and specialized training is essential to prevent errors or shortcomings that could endanger patients' lives or health. Adequate training increases mental health nurses' self-confidence in clinical practice, reduces stress, and promotes career advancement. Most importantly, it enables them to provide psychoeducation and medication education, assist in medication decisions, and support overall medication adherence.

It is crucial to raise awareness among educational institutions and clinics about the importance of educating nurses and mental health professionals on the adverse effects of antipsychotic medications. This can be achieved through extensive large-scale research in each country, along with investments in developing assessment tools for nurses and providing ongoing education.

The incorporation of extensive psychopharmacology instruction within nursing curricula constitutes a vital priority, guaranteeing that nurses acquire the essential expertise to manage intricate medication protocols effectively. Additionally, the structured deployment of continuing education initiatives centered on antipsychotic safety highlights the need for sustained professional development, enabling nurses to stay abreast of advancing best practices. These progressive approaches bear considerable significance for nursing education, policy development, and subsequent research endeavors, collectively contributing to the improvement of patient care quality and safety through evidence-based clinical decision-making.

It is crucial to raise awareness among educational institutions and clinics about the importance of educating nurses and mental health professionals on the adverse effects of antipsychotic medications. This can be achieved through extensive large-scale research in each country, along with investments in developing assessment tools for nurses and providing ongoing education.

## Use of AI tools declaration

The authors declare they have not used Artificial Intelligence (AI) tools in the creation of this article.
